# Association between Vegetable Consumption and Blood Pressure, Stratified by BMI, among Chinese Adolescents Aged 13–17 Years: A National Cross-Sectional Study

**DOI:** 10.3390/nu10040451

**Published:** 2018-04-05

**Authors:** Yide Yang, Bin Dong, Zhiyong Zou, Shuo Wang, Yanhui Dong, Zhenghe Wang, Jun Ma

**Affiliations:** 1 Department of Preventive Medicine, Medical College of Hunan Normal University, Changsha 410013, China; yangyide@bjmu.edu.cn; 2 Institute of Child and Adolescent Health, School of Public Health, Peking University Health Science Center, Beijing 100191, China; bindong@bjmu.edu.cn (B.D.); harveyzou2002@bjmu.edu.cn (Z.Z.); 1311110135@bjmu.edu.cn (S.W.); dongyanhui@bjmu.edu.cn (Y.D.); wangzhh2014@bjmu.edu.cn (Z.W.)

**Keywords:** blood pressure, obesity, adolescent, vegetables, body mass index

## Abstract

The association between vegetable intake and blood pressure (BP) in adolescents is still inconsistent, and the description of the recommended daily vegetable consumption is abstract and nonfigurative. Here we aimed to investigate the association between vegetable consumption and BP and further look for a simple way to describe a satisfactory level of daily vegetable consumption for adolescents. We recruited 18,757 adolescents, aged 13–17 years, from seven provinces in China in 2013. A standard physical examination, including height, weight and BP was conducted. Information regarding vegetable intake was collected by questionnaire, and one serving of vegetables was defined as the size of an adult’s fist. Multivariable linear and logistic regression models were used for analysis after adjusting for covariates. Approximately 12.2%, 38.0%, 28.7%, and 21.1% of the adolescents reported daily vegetable consumption of <1, 1~2, 2~3, and ≥3 servings, respectively. Adolescents whose daily vegetable consumption was ≥3 servings showed a lower risk of high blood pressure (HBP) (OR = 0.74, 95%CI: 0.58~0.94, *p* = 0.013) compared to those with daily vegetable consumptions of < 1 serving. When stratified by body mass index (BMI), in overweight adolescents, participants with 2~3 or ≥3 servings/day had an OR of 0.66 (95%CI: 0.45~0.97) or 0.63 (95%CI: 0.42~0.95) compared with the reference group. Daily vegetable intake of at least three servings (three adult’s fists) is associated with a lower HBP risk in adolescents, which leads to a simple message: “consuming at least three fists of vegetables every day will improve your blood pressure profile”.

## 1. Introduction

High blood pressure (HBP) is a leading risk factor of global disease burden, especially in Asia [1]. It is reported that HBP in childhood could track into adulthood [2]. Hence, HBP has become an important public health issue, and its early prevention is possible [3]. Dietary behavior, as a modifiable factor of cardiovascular disease, is crucial for the development of early HBP prevention and intervention. Vegetables are an important source of vitamins, minerals and fibers which could benefit cardiovascular health [4]. It has been also reported that consumption of vegetables is associated with a low risk of hypertension in adults [5]. However, in children and adolescents, inconsistent results have been reported [6]. For example, Mellendick reported that a higher total intake of vegetables was associated with a lower systolic blood pressure (SBP) [7]. However, Damasceno [8] and Mahfouz [9] did not find an inverse association between vegetable consumption and HBP. Furthermore, the impact of vegetable intake on blood pressure (BP) was always combined with that of fruit [10], and the independent effect of vegetable intake on BP is still unclear.

Currently, the dietary guidelines for vegetable intake are mostly based on studies in adults, and for adolescents, there is no consensus regarding daily vegetable consumption [6]. Different from adolescents, adults have greater life experience and have accumulated more complex environmental effects; hypertension in adults could be largely attributed to the aging process and stiffness of arterial trees [11]. In addition, for adolescents, high blood pressure (HBP) is usually defined as a systolic blood pressure (SBP) or (diastolic BP) DBP higher than the age-, sex- and height-specific 95th percentile. Hence, the influence of vegetable consumption on HBP in childhood will be, at least partially, different from that in the adult population. Additionally, the abstract description of the recommended daily vegetable intake, such as 300~500 g daily in adults [12], also limits its utility when interventions and health education are conducted.

In this study, we hypothesized that a higher daily consumption of vegetables would be associated with a lower risk of HBP in adolescents. The fist-size serving, which is easy to understand, was used to describe vegetable intake servings (Figure S1). In addition, using a simple approach to measure vegetable intake will help to estimate the appropriate daily vegetable intake to improve adolescents’ BP profiles. These results have the potential to aid in the development of health education and interventions for reducing risk of HBP and related cardiovascular diseases.

## 2. Materials and Methods

### 2.1. Study Design

A school-based cross-sectional survey was conducted in September 2013, and Chinese children and adolescents from seven provinces (Shanghai, Guangdong, Hunan, Liaoning, Ningxia, Tianjin and Chongqing) were investigated. The survey used a standardized and uniform protocol in all selected schools of the seven provinces. The multistage cluster random sampling method was adopted to recruit the representative sample of children and adolescents, and the details of this have been described in a previous study [13].

In this study, 18,757 adolescents, aged 13–17 years (9188 boys and 9569 girls), were recruited to investigate the relationship between vegetable consumption and BP. This study was approved by the Ethic Committee of Peking University Health Science Center (No. IRB0000105213034). Written informed consents were provided by participants, and also by their parents, voluntarily.

### 2.2. Questionnaire

Data regarding demographic information, consumption of fruit and vegetables, and time spent on physical activity were collected with a standard questionnaire [14]. The frequency (days) and amount (servings, 1 serving ≈ 200 g) of fruit and cooked vegetables (not including pickles), separately, consumed in the past 7 days were investigated [13]. The average daily consumption of vegetables was calculated as follows: average daily vegetable intake = (frequency × (vegetable intake on each of the days))/7. A similar equation was used to calculate the daily fruit intake as well. For a better understanding of the vegetable and fruit intake, one serving (about 1 cup, ≈200 g) [15] was defined as the size of an adult’s closed fist, and a fist figure was provided to explain the amount visually (Figure S1). The level of vegetable or fruit consumption was categorized in groups of “<1 serving/day”, “1–2 servings/day (≥1 and <2 servings/day)”, “2–3 servings/day (≥2 and <3 servings/day)”, and “≥3 servings/day”. The level of physical activity was measured by the self-reported daily time spent on physical activity in the past 7 days, with the options of “<1 h”, “1–2 h (≥1 h and <2 h)”, “2–4 h (≥2 h and <4 h)”, “≥4 h”, and “not sure”. Smoking and alcohol intake was defined as having occurred in the previous 30 days.

### 2.3. Physical Examination

Anthropometric measurements, including height, weight and BP, were measured in accordance with standard protocols [16,17]. Height and weight were measured twice, and the mean value was used for analysis, and participants were asked to only wear light clothes and stand straight without shoes. Weight was measured to the nearest 0.1 kg using a lever weight scale (model RGT-140, Changzhou, China). Height was measured to the nearest 0.1 cm using a stadiometer (model TZG, Shanghai, China).

BMI (body mass index) was calculated by weight (kg) divided by height (m) squared (kg/m^2^). In accordance with the “BMI Reference for Screening Overweight and Obesity in Chinese School-age Children” developed by Working Group on Obesity in China, participants were defined as overweight if their BMI was ≥the age- and sex-specific cut-off values (85th percentile), which includes individuals being overweight and obese; participants were considered non-overweight if their BMI was <the age- and sex-specific cut-off values (85th percentile) (Table S1) [18].

BP was calculated by averaging three measurements taken on one single visit. It was measured three times with a 5-min time interval between measurements. BP was measured according to the recommendation of the National High Blood Pressure Education Program Working Group for Children and Adolescents, using an auscultation mercury sphygmomanometer with an appropriate cuff size for children. BP measurements were taken at least 5 min after resting. SBP was defined as the onset of the “tapping” Korotkoff sound (K1), and diastolic BP (DBP) was defined as the fifth Korotkoff sound (K5). HBP was defined as SBP and/or DBP ≥ the age-, sex- and height-specific 95th percentile references [19].

### 2.4. Statistical Analysis

Data analysis was performed using SPSS for Windows (version 20.0, SPSS Inc., Chicago, IL, USA). We tested the normality with the Kolmogorov-Smirnov test and found that continuous variables did not conform to normal distribution. Therefore, continuous variables were described as medians and inter quartile ranges (IQR), and categorical variables were described as frequencies. Comparisons of continuous variables were conducted with the Mann–Whitney U test and categorical variables by the Chi square test. BPs, including SBP and DBP, were transformed into age-, sex- and height- adjusted BP *z* scores, in accordance with the recommendations of the National High Blood Pressure Education Program Working Group for Children and Adolescents [19]. Multivariable linear regressions were used to analyze the associations between vegetable consumption and BP *z* scores. Multivariable logistic regression was used to estimate the association between vegetable consumption and HBP when covariates, including age, province, sex, BMI, physical activity and fruit consumption, were adjusted for. Since there is plenty of evidence showing that obesity is closely associated with both blood pressure [17] and vegetable intake [20], we stratified by BMI groups to exclude the influence of obesity.

## 3. Results

### 3.1. General Characteristics of Participants

A total of 18,757 adolescent participants were included in the analysis, 9188 (49.0%) were boys and 9569 (51%) were girls, and 12.2%, 38.0%, 28.7%, and 21.1% of the participants reported consuming vegetables in quantities of <1, 1~2, 2~3, and ≥3 servings daily, respectively. In total, 6.2% of the participants were classified as having HBP, and 17.4% of the participants were overweight. In total, 25% of the participants had <1 serving/day of fruit, and 37.7% had participated in physical activity <1 h/day in the past week. In total, 1% of the participants smoked, and 4.8% had drunk alcohol in the past 30 days (Table 1).

### 3.2. Association between Vegetable Consumption and BP z Score

The associations between vegetable consumption and BP are shown in Table 2 by BMI group. In both BMI groups, the SBP *z *score was inversely associated with vegetable consumption (*p* < 0.015). Compared with non-overweight adolescents, the magnitude of the association in participants who were overweight was more profound, with coefficients of −0.016 and −0.027, respectively, in adjusted models. A similar pattern was observed for the corresponding association for the DBP *z* score, though the relationship was only statistically significant relative to the total group of participants.

### 3.3. Risk of High BP in Adolescents in Various BMI Groups and Vegetable Consumption Groups

In the population as a whole, compared with the lowest daily vegetable consumption group, individuals in the group with three servings or more of vegetables daily showed a significant association with lower odds of HBP (OR = 0.73, 95%CI = 0.57~0.93, *p* = 0.009) with age, sex, province, BMI, physical activity, fruit consumption, smoking and alcohol intake as covariates (Table 3).

**Figure 1:**
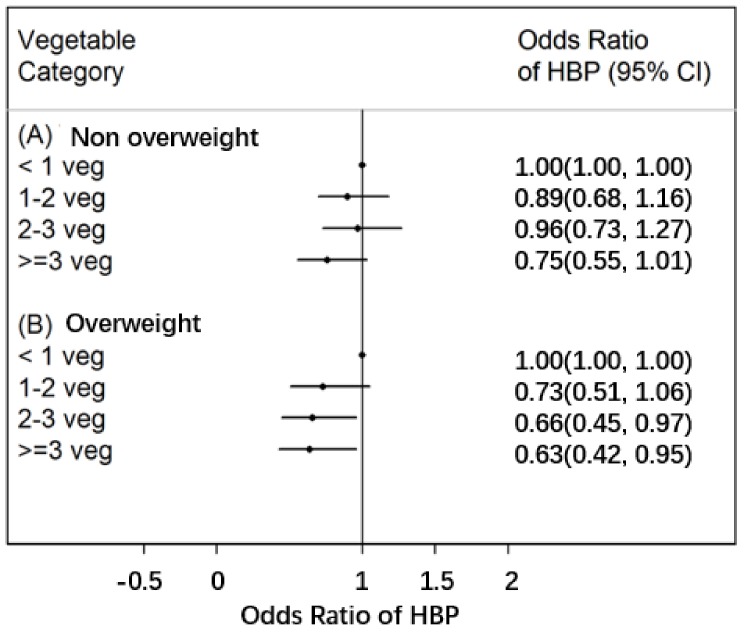
Associations between vegetable consumption and the risk of HBP stratified by BMI (non-overweight group and overweight group). HBP: high blood pressure. Values are adjusted for age, sex, province, BMI, physical activity, fruit consumption, smoking and alcohol intake. Overweight is defined as BMI ≥ the age- and sex-specific cut-off values (85th percentile) which includes individuals who are overweight and obese; non-overweight is defined as BMI < the age- and sex-specific cut-off values (85th percentile) which includes individuals who are normal weight and underweight.).

The analyses were further performed in different BMI groups, with the least daily vegetable consumption group being the reference group. In the overweight group only, there was a statistically significant relationship between the consumption of two or more servings of vegetables per day and a decline in the odds of HBP, with OR of 0.66 (95%CI = 0.45~0.97) for the 2–3 servings group and an OR of 0.63 (95%CI = 0.42~0.95) for the more than 3 servings group (Figure 1). 

## 4. Discussion

In our study, which included 18,757 adolescents aged 13–17 years of age, a high level of vegetable intake was related to a decreased BP level. Further, in the total population, consuming more than three servings (three adult’s closed fists, or about 600 g) of vegetables was associated with a lower HBP risk. When stratified by BMI, in overweight adolescents, participants consuming ≥ two servings/day had a relatively lower risk of HBP compared with the reference group. These associations persisted when confounding factors, including fruit consumption, physical activity, smoking and alcohol intake were adjusted for. In addition, a simple approach was provided to describe the amount of vegetable intake, which could benefit health education and interventions aimed at reducing the HBP burden and related cardiovascular disease risk in this population.

### 4.1. Vegetable Consumption in Adolescents

Insufficient consumption of vegetables among children and adolescents is common in Asian countries. A daily consumption of vegetables of 300~500 g is recommended for adults and adolescents in China, which means 2~3 servings everyday (1 serving ≈ 200 g) [12]. However, less than half of the participants in our study meet this recommendation, and about one in eight adolescents consumed hardly any vegetables. In line with our results, a study involving five Southeastern countries reported that about 13.8% of adolescents aged 13–15 years did not have vegetables every day [21]. A study conducted in children from Thailand reported similar results, with 65.8% eating less than two servings of vegetables (one serving meant 1/2 a cup of cooked vegetables, or one cup of salad vegetables or one medium potato in that study) per day [22]. Compared with Asian adolescents, more vegetables are consumed by their peers in Western countries. For example, Krupp and colleagues also reported that the fruit and vegetables consumption in German children was 407 g/day in boys and 388 g/day in girls [23].

Since dietary behaviors in adolescence could track into the adulthood [24], it is of great significance to change unhealthy dietary behaviors in early life, and relevant interventions could encourage healthy lifestyles in adulthood.

### 4.2. Association between Vegetable Consumption and HBP in Adolescents

Previous studies have reported the association between vegetable consumption and BP, as well as HBP risk, in adolescents [8,9,10,25]. However, many of those studies reported the combined effect of fruit and vegetables, and only frequency [9,25,26], rather than both frequency and amount [9,10], of daily vegetables intake was investigated. Shi and colleagues reported that every 100 g/day less in fruit and vegetable intake was associated with a 0.4 mmHg increase in both SBP and DBP [10]. Their findings were also supported by several lifestyle interventions aimed at improving cardiovascular health, including the DASH diet [27]. In our study, an increase of one vegetable serving (about 200 g) was related to a decrease of 0.012 in the SBP *z* score (about 0.14 mmHg), and a daily consumption of more than three servings (about 600 g) was associated with lower odds of having HBP. In particular, in overweight adolescents, the HBP risk declined by more than 34% in those consuming two or more servings of vegetables every day. Although the underlying mechanisms of how vegetable intake benefits BP is not fully elucidated [6], studies have revealed that vegetables are rich in fiber, folic acid, potassium [23], and phytochemicals (such as antioxidants) [28]. These nutrients are suggested to be beneficial for the BP profile [29].

### 4.3. Implications

Our findings could be helpful for clinical practices and public health services for adolescents. First, the better BP profile observed in subjects with higher intake of vegetables confirms that a diet rich in vegetables could be effective for BP control. Additionally, consuming more than three servings of vegetables daily can be recommended for adolescents, though those who are overweight may benefit by consuming more than two servings of vegetables. Moreover, in our study, one serving was described as the size of an ordinary adult’s fist, which is useful and direct, especially in educational settings. A simple message of “consuming at least three fists of vegetables every day will improve your blood pressure profile” could be easy for non-medical professionals, including parents and adolescents, to understand, and could be applied in interventions and health education.

### 4.4. Limitations

However, there were also limitations in the present study. First, this was a cross-sectional study and only associations, rather than casual relationships or effects of dietary behavior on BP, could be generated. A randomized controlled trial is desired to confirm our results. Second, other important factors, such as family medical history, salt intake and calorie intake, were not available in the present study. Considering the high salt intake of Chinese children [3], whether the salt intake modulated the association between BP and vegetables intake is worth studying in the future. Third, the present study focused on 13–17 years old adolescents, thus its generalization to young children is unclear. Finally, we cannot identify what vegetables were consumed by the participants, and different vegetables may have various influences on BP level. A study with more information regarding confounding factors and vegetable consumption is desired in the future. Fourth, the dietary data recall of 7 days may not represent long-term dietary habits; thus a future study with a questionnaire regarding long-term habits should be conducted.

## 5. Conclusions

In conclusion, the present study found that daily consumption of at least three servings (three adult’s closed fists, or about 600 g) of vegetables was associated with a lower HBP risk in adolescents, particularly in overweight individuals. A message of “consuming at least three fists of vegetables every day will improve your blood pressure profile” would be simple and useful for health education and interventions aimed at improving adolescents’ cardiovascular health.

## Figures and Tables

**Table 1:** The characteristics of the Chinese adolescents according to vegetable intake.

Variables	Vegetable Consumption Group										*p*
	<1 Servings/Day		1~2 Servings/Day		2~3 Servings/Day		≥3 Servings/Day		Total		
	*n*	Freq/Median	*n*	Freq/Median	*n*	Freq/Median	*n*	Freq/Median	*n*	Freq/Median	
N	2285	12.20%	7136	38.00%	5377	28.70%	3959	21.10%	18,757	100.00%	
**Sex**											
Boys	1101	48.20%	3443	48.20%	2577	47.90%	2067	52.20%	9188	49.00%	<0.001
Girls	1184	51.80%	3693	51.80%	2800	52.10%	1892	47.80%	9569	51.00%	
Age, years	2285	15 (13,16)	7136	15 (13,16)	5377	15 (13,16)	3959	15 (13,16)	18,757	15 (13,16)	<0.001
Height (cm)	2285	161.9 (156.9,168.3)	7136	162.5 (157.2,169)	5377	162.5 (157.3,169.1)	3959	163.1 (157.9,169.4)	18,757	162.6 (157.3,169)	<0.001
Weight (kg)	2285	52.2 (46.7,59.7)	7136	53 (47,60.3)	5377	53 (46.7,61)	3959	53 (46.6,61.4)	18,757	52.9 (46.9,60.6)	0.083
BMI (kg/m^2^)	2285	19.8 (18.0,22.0)	7136	19.8 (18.2,22.1)	5377	19.8 (18.1,22.3)	3959	19.7 (17.9,22.3)	18,757	19.8 (18.1,22.2)	0.283
SBP (mmHg)	2285	110 (101,118)	7136	110 (100,120)	5377	110 (100,119)	3959	110 (100,119)	18,757	110 (100,119)	0.385
DBP (mmHg)	2285	70 (62,74)	7136	70 (62,73)	5377	70 (62,74)	3959	70 (61,73)	18,757	70 (62,73)	0.009
**HBP group**											0.521
Non-HBP	2130	93.20%	6711	94.00%	5039	93.70%	3719	93.90%	17,599	93.80%	
HBP	155	6.80%	425	6.00%	338	6.30%	240	6.10%	1158	6.20%	
**Fruit intake** (serving/day)											
<1	1134	50.80%	1913	27.10%	1031	19.40%	549	14.00%	4627	25.00%	<0.001
1~2	778	34.80%	3933	55.80%	2803	52.70%	1614	41.20%	9128	49.30%	
2~3	231	10.30%	925	13.10%	1111	20.90%	1003	25.60%	3270	17.70%	
≥3	90	4.00%	283	4.00%	378	7.10%	749	19.10%	1500	8.10%	
**BMI groups**											
Non-overweight	1910	83.60%	5969	83.60%	4418	82.20%	3201	80.90%	15,498	82.60%	0.001
Overweight	375	16.40%	1167	16.40%	959	17.80%	758	19.10%	3259	17.40%	
**Physical activity** (hour/day)											
<1	925	41.40%	2911	41.60%	1897	35.90%	1200	31.00%	6933	37.70%	<0.001
1~2	719	32.20%	2259	32.30%	2006	37.90%	1400	36.10%	6384	34.70%	
2~4	286	12.80%	939	13.40%	719	13.60%	678	17.50%	2622	14.20%	
≥4	101	4.50%	371	5.30%	340	6.40%	354	9.10%	1166	6.30%	
not sure	205	9.20%	521	7.40%	326	6.20%	245	6.30%	1297	7.00%	
**Smoking**											
No	2201	98.6%	6917	99.1%	5209	99.0%	3841	98.8%	18,168	99.0%	0.069
Yes	32	1.4%	60	0.9%	50	1.0%	47	1.2%	189	1.0%	
**Alcohol intake**											
No	2094	93.7%	6672	95.7%	5005	95.4%	3686	95.0%	17,457	95.2%	0.002
Yes	140	6.3%	302	4.3%	242	4.6%	192	5.0%	876	4.8%	

Notes: BMI, body mass index. SBP, systolic blood pressure; DBP, diastolic blood pressure; HBP, high blood pressure; Freq, Frequency; SD, standard deviation; Continuous variables are described as medians and inter quartile ranges (IQR) and compared by Mann-Whitney U tests. Overweight is defined as BMI ≥ the age- and sex-specific cut-off values (85th percentile) which includes individuals who are overweight and obese; non-overweight is defined as BMI < the age- and sex-specific cut-off values (85th percentile) which includes individuals who are normal weight and underweight.

**Table 2:** Association between vegetable consumption and BP *z* score among 13–17-year-old adolescents, stratified by BMI.

BMI groups	BP(mmHg)	Model	Coefficient	SE	*p*
Total	SBP *z* score	Crude model	−0.012	0.005	0.015
		Adjusted model	−0.018	0.005	<0.001
	DBP *z* score	Crude model	−0.005	0.003	0.132
		Adjusted model	−0.009	0.004	0.018
Non-overweight	SBP *z* score	Crude model	−0.015	0.005	0.004
		Adjusted model	−0.016	0.006	0.003
	DBP *z* score	Crude model	−0.007	0.004	0.082
		Adjusted model	−0.008	0.004	0.041
Overweight	SBP *z* score	Crude model	−0.023	0.012	0.049
		Adjusted model	−0.027	0.012	0.029
	DBP *z* score	Crude model	−0.011	0.008	0.185
		Adjusted model	−0.012	0.009	0.154

Crude model adjusted for age, province and sex. Adjusted model adjusted for age, province, sex, BMI, physical activity, fruit consumption, smoking and alcohol intake. BMI, body mass index; SBP, systolic blood pressure; DBP, diastolic blood pressure; SE, standard error; overweight is defined as BMI ≥ the age- and sex-specific cut-off values (85th percentile) which includes individuals who are overweight and obese; non-overweight is defined as BMI < the age- and sex-specific cut-off values (85th percentile) which includes individuals who are normal weight and underweight.

**Table 3:** Association between vegetable consumption and high blood pressure in 13–17-year-old adolescents.

Vegetable Consumption	non-HBP		HBP		OR *	*p*
	*n*	Freq (%)	*n*	Freq (%)		
<1 serving/day	2130	12.10	155	13.39	1 (ref)	
1–2 servings/day	6711	38.13	425	36.70	0.85 (0.69–1.05)	0.132
2–3 servings/day	5039	28.63	338	29.19	0.86 (0.69–1.07)	0.180
≥3 servings/day	3719	21.13	240	20.73	0.73 (0.57–0.93)	0.009

* Adjusted for age, province, sex, BMI, physical activity, fruit consumption, smoking and alcohol intake; BMI, body mass index; HBP, high blood pressure; Freq, Frequency; HBP was defined as SBP and/or DBP ≥ the age-, sex- and height-specific 95th percentile references, in accordance with the recommendations of the National High Blood Pressure Education Program Working Group for Children and Adolescents.

## Supplementary Materials

The following are available online at http://www.mdpi.com/2072-6643/10/4/451/s1, Figure S1: The size of one serving of fruit or vegetable (as the size of a fist of ordinary adult) (The ratio is 1 to 1), Table S1: Body mass index reference for screening overweight and obese individuals in Chinese school-age children (kg/m^2^).
