# Supplementary material for: Association between Vegetable Consumption and Blood Pressure, Stratified by BMI, among Chinese Adolescents Aged 13–17 Years: A National Cross-Sectional Study
Source: Nutrients. 2018 Apr 5;10(4):451. doi: 10.3390/nu10040451 (PMC5946236; doi:10.3390/nu10040451)
Supplement: Supplementary file 1 [file nutrients-10-00451-s001.pdf]

Supplementary Material

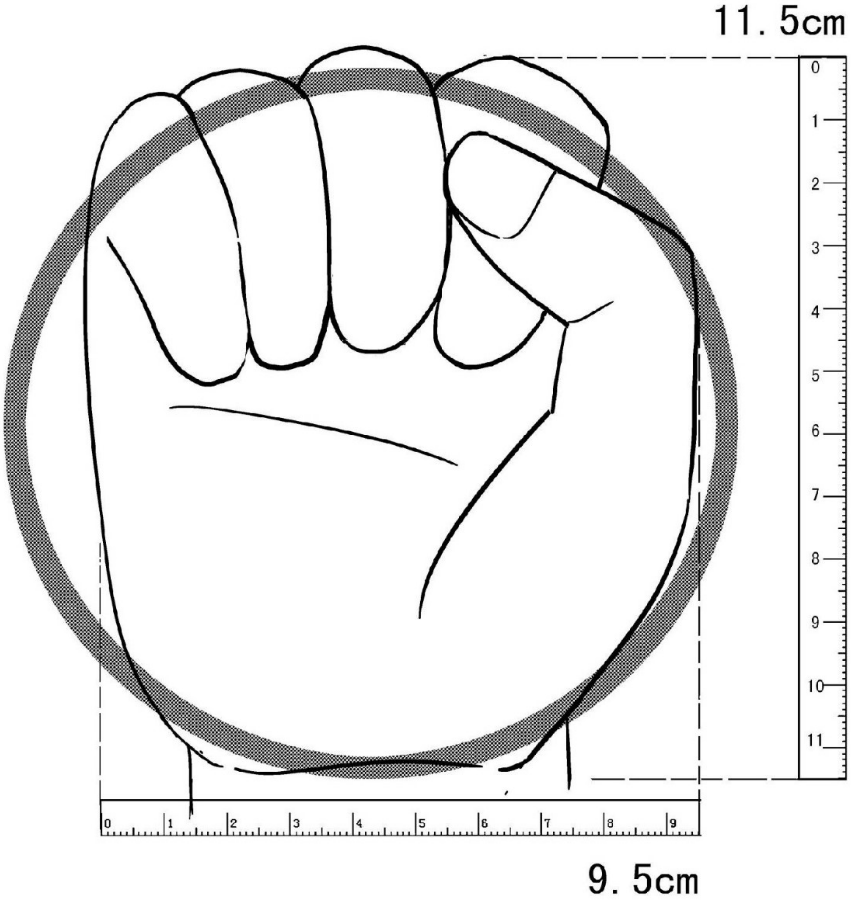

Figure S1. The size of one serving of fruit or vegetable (as the size of a fist of ordinary adult).

Table S1. Body mass index reference for screening overweight and obesity in Chinese school-age children (kg/m^2).

Age (years)	Boys		Girls	
	overweight	obesity	overweight	obesity
13～	21.9	25.7	22.6	25.6
14～	22.6	26.4	23.0	26.3
15～	23.1	26.9	23.4	26.9
16～	23.5	27.4	23.7	27.4
17～	23.8	27.8	23.8	27.7

Ref: Ji CY, Working Group on Obesity in C (2005) Report on childhood obesity in China (1)--body mass index reference for screening overweight and obesity in Chinese school-age children. *Biomedical and environmental sciences: BES* **2005**, *18*, 390–400.
